# Angiogenic and Inflammatory Alterations of Endometriotic Lesions in a Transgenic Animal Experimental Model With Loss of Expression of PPAR-Alpha Receptors

**DOI:** 10.7759/cureus.30290

**Published:** 2022-10-14

**Authors:** Vasilios Pergialiotis, Nikolaos Zarkadoulas, Kallirroi Goula, Maximos Frountzas, Fotino Antoniadou, Dimitrios Dimitroulis, Dimitrios Vlachos, Aggeliki Papapanagiotou, Christos Verikokos, Despoina N Perrea, Konstantinos Kontzoglou

**Affiliations:** 1 First Department of Obstetrics and Gynecology, National and Kapodistrian University of Athens, Athens, GRC; 2 Laboratory of Experimental Surgery and Surgical Research N.S. Christeas, National and Kapodistrian University of Athens School of Medicine, Athens, GRC; 3 Department of Pathology, Alexandra Hospital, Athens, GRC; 4 First Propaedeutic Department of Surgery, Hippocration General Hospital, National and Kapodistrian University of Athens School of Medicine, Athens, GRC; 5 Second Department of Surgery, National and Kapodistrian University of Athens School of Medicine, Athens, GRC; 6 First Department of Obstetrics and Gynecology, National and Kapodistrian University of Athens School of Medicine, Athens, GRC; 7 Department of Biological Chemistry, National and Kapodistrian University of Athens School of Medicine, Athens, GRC; 8 Second Department of Surgery, Laikon Hospital, National and Kapodistrian University of Athens School of Medicine, Athens, GRC; 9 Laboratory of Experimental Surgery and Surgical Research, National and Kapodistrian University of Athens School of Medicine, Athens, GRC

**Keywords:** 129s4-pparatm1gonz/j, angiogenesis, mice, peroxisome proliferator, ppar-alpha, endometriosis

## Abstract

Introduction: Peroxisome proliferator-activated receptors (PPARs) have been proposed as a medical treatment against endometriosis in preclinical and clinical studies. Their effect seems to be triggered through the suppression of angiogenesis. In the present study, we used a transgenic animal model with a loss of expression of PPAR-alpha receptors to examine their effect on the course of surgically induced endometriotic lesions.

Methods: Ten C57BL/6 mice that served as controls and 10 B6;129S4-PPARa^tm1Gonz/J t^ transgenic mice characterized by absolute loss of expression of PPAR-alpha receptors were used for induction of endometriosis with a previously described surgical technique.

Results: Five animals (50%) exhibited abundant endometriotic crypts in the control group whereas only one (10%) animal in the transgenic experimental group had a similar pathological image. Neo-vascularization significantly differed among the two groups (p=0.034) favoring the control group as it was extremely limited in half of the PPAR-alpha null animals. The median inflammation score was 2.5 (1-4) in the P B6;129S4-PPARa^tm1Gonz/J^ group, whereas it was minimal, 1 (0-2), in the C57BL/6 group. However, these differences were not statistically significant (p=0.101). The fibroblastic activity was also very limited in the PPAR-alpha-deficient model, whereas animals belonging to the control group exhibited an intermediate increase of this index (p=0.022).

Conclusion: Surgically induced endometriotic implants in animals with loss of expression of PPAR-alpha receptors exhibit significant differences in their pathology compared to lesions induced in control animals. This information suggests that PPAR-alpha receptors have a significant impact on the course of the disease, indicating that they may serve as potential targets for future medical therapies.

## Introduction

Endometriosis is an autoimmune hormone-dependent disease that exerts a detrimental impact on the quality of life of women of reproductive age, with an incidence of 4-10% [1]. Symptoms vary significantly ranging from cases that are totally asymptomatic, which are frequently observed during a routine transvaginal ultrasound or intraoperatively in women offered a procedure for other pathologic entities, to cases that report pelvic pain, dyspareunia, infertility, and in the most severe forms dyschezia, blood loss during defecation and symptoms related to an extraperitoneal disease such as catamenial pneumothorax/hemothorax that is accompanied with hemoptysis and in rare cases of cerebral endometriosis with headaches and gait disturbances [2].

Although endometriosis constitutes a quite common gynecological disease, its pathophysiology is not yet utterly elucidated. Various mechanisms that may trigger its onset have been studied from time to time in the international literature, including immunological, endocrine, genetic, and finally epigenetic factors [3], and in the latest theory Koeninckx et al. support that its pathophysiology is multimodal [4]. Specifically, it seems that endometriosis is triggered by a complex interplay that involves factors associated with the occurrence of oxidative stress during menstruation which eventually affects the transcription of several genes that have been implicated in the formation of endometriotic lesions [3].

The impact of peroxisome proliferator-activated receptors (PPARs) in endometriosis has been previously described by several researchers who observed that activation of PPAR-gamma receptors may inhibit the growth and survival of human endometriotic cells through suppression of estrogen biosynthesis [5,6]. Recent studies have shown that combined activation of PPAR-alpha and PPAR-gamma with resveratrol may effectively reduce the size of endometriotic implants in experimental models of endometriosis [7]. This effect seems to be triggered by lipidomic alterations and is supported by clinical data which have previously designated the existence of altered lipid profiling in endometriotic lesions [8,9]. Taking this information into consideration it may be speculated that PPAR-alpha receptors may also act as potential regulators of endometriosis [10]. Although mostly expressed in the liver, PPAR-alpha proteins are also observed in endothelial cells and immune-type cells, including the monocyte macrophages and the endometrial tissue [11]. Oncologic research showcased that PPAR-alpha agonists seem to limit the process of angiogenesis, increasing the expression of anti-angiogenic molecules, such as thrombospondin-1 (TSP-1), gypenoside 140 (gp140), and factors involved in the cascade of protein kinases that trigger mitotic divisions of cancer cells [12]. PPAR-alpha-deficient mice seem to have diminished tumor development, an effect that is triggered by altered CYP2C9 epoxygenase expression, which is responsible for neo-vascularization.

Based on the common profile of neo-angiogenesis of endometriotic lesions and several other cancer forms, including ovarian cancer, we chose to conduct an experimental study on PPAR-alpha-deficient mice to evaluate the impact of this deficiency on the pathology analysis of surgically induced endometriotic lesions.

## Materials and methods

Animals

Two B6;129S4-PPARatm1Gonz/J mice (The Jackson Laboratory) were delivered at the Laboratory of Experimental Surgery and Surgical Research “N.S. Christeas”, Medical School, National and Kapodistrian University of Athens. Animal breeding was performed using standard procedures until a minimum number of 12 female animals of reproductive age were available. Twelve C57BL/6 mice that served as controls (Hellenic Pasteur Institute, Department of Animal Models for Biomedical Research, Greece) were delivered by that time at the laboratory and were kept for seven days in order to adapt to their new environment in atmosphere-controlled chambers (temperature 20 ± 1˚C, humidity 55 ± 5%) and under controlled light (12 hours per day) for seven days. ELVIZ 510 food pellets were utilized for full nutritional supplementation ad libitum for all animals. Food was not administered the night before surgery.

Research involving animals

Animal care, surgical procedures, and postoperative recovery complied with the Animal Research Reporting of In Vivo Experiments (ARRIVE) guidelines and were approved by the Athens University Medical School Ethics Committee (Institutional Review Board Number: 1088/2018) and the Veterinary Directorate of the Ministry of Agriculture in agreement with the EU Directive 2010/63/EU for animal experiments.

Experimental protocol and surgical procedures

Anesthesia was performed with an intraperitoneal injection of 5 mg/kg xylazine hydrochloride and 60 mg/kg ketamine. Xylazine is an analog of clonidine and an agonist at the α-2 adrenergic receptors. Ketamine is used for inducing and maintaining anesthesia, which induces a trance-like state while providing analgesia, sedation, and memory loss [13]. The anterior abdominal surface was prepared with povidone iodine solution, alcohol, and single-use sterile drapes. A vertical ventral incision of about 3 cm starting at the level of the pubic symphysis was performed. After entering the peritoneal cavity, we identified the uterine horns of the mice. One of the uterine horns was extracted and a longitudinal incision was performed to exteriorize the endometrium as previously described in our previous research [14]. A part of the specimen with a length of approximately 1 cm and an average width of 0.5 cm was used and transplanted in the pelvic cavity of the mice using a suture to ensure that the mucosa would be in direct contact with the peritoneal surface. Following auto-transplantation, 2 ml saline was instilled in the pelvic cavity in order to limit the formation of adhesions and drying of the implant. The abdominal incision was closed in layers using a mass closure 3-0 multifilament (90% glycolide, 10% L-lactide) Vicryl continuous suture, and the skin was closed with 4-0 monofilament non-absorbable, nylon continuous suture.

Postoperatively, appropriate levels of analgesia were ensured with the use of subcutaneous administration of tramadol at a dose of 0.01 mg/kg. Access to food and water was provided freely 8 hours after the operation by adopting enhanced recovery after surgery (ERAS) protocols [15]. The animals were sacrificed on postoperative day 15 taking into consideration previous observations that support this interval as the optimal [16]. An en bloc excision of the pelvic implant together with a 1 cm margin of the attached pelvic peritoneum was performed to ensure adequate specimen for inspection. Samples were fixed in 10% formaldehyde solution and histopathological analysis followed.

Histopathological analysis

Tissue specimens were fixed and preserved in a 10% solution of formaldehyde and then embedded in paraffin blocks and cut into 4 μm sections using a microtome (Histocore Multicut, Leica Biosystems®, Wetzlar, Germany). Histopathologic examination was blindly conducted by two pathologists. Standard hematoxylin-eosin (H&E) staining was applied. In addition, CD-31 and CD-34 immunohistochemistry was applied to evaluate the amount of neovascularization by targeting the presence of endothelial cells. Briefly, the slides were immersed in an antigen retrieval solution (Reveal - Biocare Medical, Concord, CA) for 30 seconds at 125°C using a decloaking chamber (Biocare) at 18-24 PSI. The primary antibody (CD31 and CD34) was diluted at 1/100 using Da Vinci diluent (Biocare Medical) and incubated for 60 minutes. To detect the presence of the antibody we used PromARK Mouse-on-Canine HRP-Polymer (Biocare Medical). The positivity of CD-31 and CD-34 staining was assessed in the presence of at least 10% of cells containing distinct labelling (otherwise it was considered null). Specimens were evaluated in terms of abundance of endometriotic crypts, collagen content, fibroblast activity, presence of granulocytes, and mononuclear cells and photographed. The assessment of the previously mentioned parameters was based on three novel scoring systems that originated from already described pathological scores as well as from modifications of the Ehrlich-Hunt model [17-21].

Statistical analysis

All analyses were conducted using the SPSS version 25.0 software for Macintosh (SPSS Inc., Chicago, IL, USA). Intention-to-treat analysis was performed. The number of animals that were used in the present experimental protocol is based on the research equation method (PMID: 16391426, 24250214). Continuous variables were presented as mean and standard deviation (±SD) and as median (range) values. The normality of the distributions was assessed with Kolmogorov-Smirnov’s test and graphical methods. Quantitative variables were presented with absolute and relative frequencies. Student’s t-test or non-parametric Mann-Whitney test was utilized to compare means between groups. The comparisons of proportions took place using the chi-square and Fisher’s exact tests. All reported p-values are two-tailed. Statistical significance was set at p <0.05.

## Results

Animal characteristics

Overall, 24 female mice were enrolled in the present study, 12 C57Bl/6 and 12 B6;129S4-PPARatm1Gonz/J. Their average weight at the onset of the experiment was 214 g (204-221) and 208 g (202-222), respectively (p=0.280). Their average age was also comparable. All animals underwent the predetermined procedure on the same day and were euthanized 15 days later.

Perioperative outcomes

Intraoperatively, one bleeding episode from the uterine horn occurred in one C57Bl/6 animal that was corrected by means of suture placement; however, the animal succumbed within the next few minutes. Another animal had hypoplastic uterine horns and the procedure was abandoned due to the insufficient size of the specimen which could not ensure the formation of a proper endometriotic implant. In the B6;129S4-PPARatm1Gonz/J, two intraoperative deaths were noticed that were attributed to ketamine overdose that led to cardiopulmonary arrest. The postoperative course was uneventful in the whole remaining series of animals.

Animal characteristics at re-operation

The animal weight did not differ among the two groups at re-operation. Specifically, the mean weight of PPAR-alpha-deficient mice was 240 (231-246) g and of controls 243 (232-254) g (p=0.389). Macroscopic evaluation of the pelvic peritoneum revealed the presence of a cystic lump at the site of surgically implanted parts of the uterine horns in all cases in the C57BL/6 group and eight cases in the B6;129S4-PPARatm1Gonz/J group. The mean lump size in the PPAR-alpha-deficient group was 1.2 (0.8, 1.5) and in the control group 1.3 (0.8 - 1.7), which was not statistically different (p=0.149). In two animals of the control group, a dense tissue was observed that was owed to extensive adhesion formation in the region. This tissue was also removed and sent for pathology analysis. Extensive adhesions in the pelvic cavity and/or upper abdominal cavity of the animals were not noticed.

Pathology analysis

**Figure 1:**
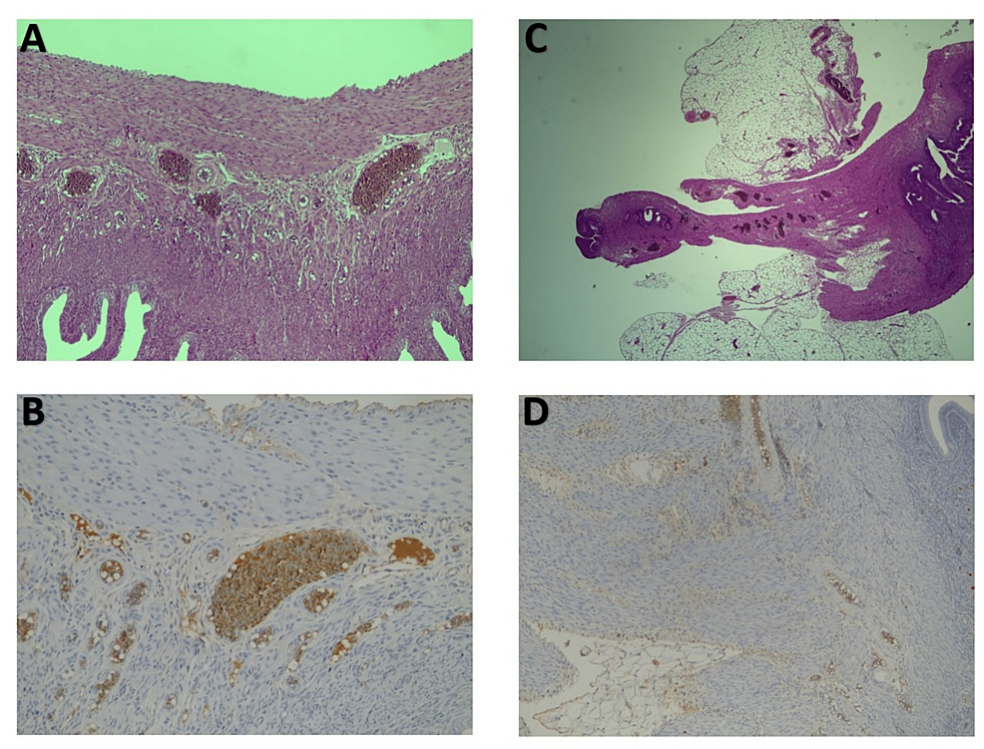
Endometriotic lesions A) H&E 20x abundant vascularization in C57BL/6 mice, B) CD34 immunohistochemistry 20x depicts significant neo-angiogenesis in the control group, C) H&E 10x depicts significant vascularization (brown staining), D) CD34 immunohistochemistry 10x indicates the presents of several microvessels (brown staining). H&E, hematoxylin and eosin.

The epithelium that covered the cystic spaces was columnar as described in our previous experiments indicating the successful achievement of endometriotic implants in both groups of animals. Endometrial stroma was not detected; however, this is normal for experimental models of endometriosis as the uterus of small rodents is mainly composed of loose fibrous tissue that is very different from that of normal human endometrial stroma which is hypercellular. The number of endometriotic crypts was significantly more prevalent in the control group indicating that PPAR-alpha receptors may be a determinant factor that contributes to the pathophysiology of endometriosis (Table 1, Figure 1).

**Table 1:** Pathology analysis of endometriotic lesions among the two groups. Each variable is graded in a five-scale score (0-4) and the number of animals assigned to each score per group is depicted in each cell.

	C57BL/6	129S4-PPARa^tm1Gonz/J^	p-Value
Neovascularization			
0	0	3	0.034
+1	0	2	
+2	2	3	
+3	5	2	
+4	3	0	
Endometriotic crypts			
0	0	0	0.059
+1	1	4	
+2	2	3	
+3	4	2	
+4	3	1	
Collagen content			
0	0	3	0.136
+1	2	3	
+2	3	3	
+3	1	1	
+4	4	0	
Fibroblast activity			
0	2	2	0.022
+1	4	0	
+2	4	3	
+3	0	4	
+4	0	1	
Inflammation			
0	2	0	0.101
+1	4	2	
+2	4	3	
+3	0	3	
+4	0	2	

**Figure 2:**
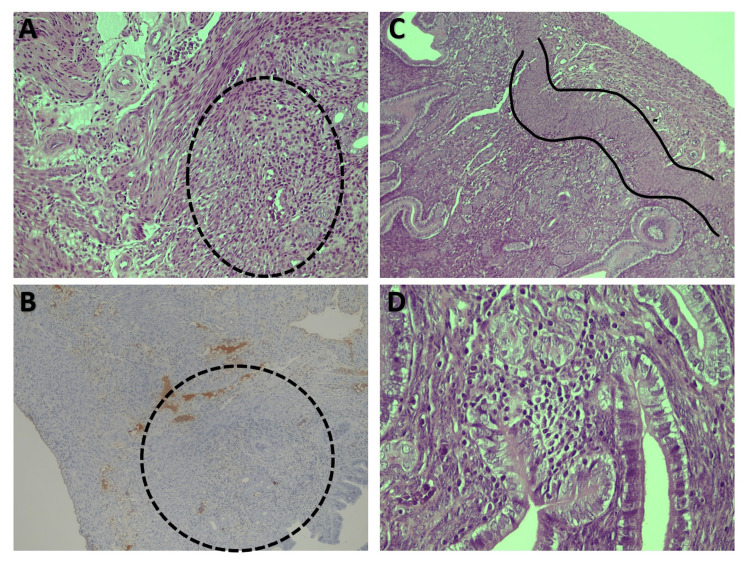
Endometriotic lesions - inflammation A) H&E, 20x presence of inflammatory cells in 129S4-PPARatm1Gonz/J mice (rounded circle), B) CD31 immunohistochemistry 10x depicts significant inflammation in the encircled area in 129S4-PPARatm1Gonz/J mice, C) 10x and D) 40x abundant crypt formation in C57BL/6 mice. The muscularis layer of the attached uterine horn is depicted in the isolated area in picture C. H&E, hematoxylin and eosin.

Abundant endometriotic crypts were noted in five animals (50%) of the control group (Figure 2) and only one (10%) of the 129S4-PPARatm1Gonz/J group. This effect was accompanied by significant differences in neo-vascularization (p=0.034), which was minimal in half of PPAR0-alpha null animals compared to controls which exhibited an increased process in 80% of their population.

The scoring system for inflammation was based on the evaluation of the leucocyte density of endometriotic implants and the expansion of leukocyte infiltration. We observed that the median inflammation score was 2.5 (1-4) in the 129S4-PPARatm1Gonz/J C57BL/6 group (Table 1, Figure 2), whereas the inflammation score was 1 (0-2) in the C57BL/6 group. Differences did not reach, however, statistical significance (p=0.101). To evaluate the degree of tissue fibrosis we compared the extent of collagen disarray, the abundance of tissue necrosis, and the percentage of fibroblasts in the fibrotic tissue. Significant differences were noted in fibroblast activity which was minimal in the PPAR-alpha-deficient model and of intermediate severity in the control group (p=0.022). Collagen content also differed among the two groups; however, differences did not reach the level of statistical significance (p=0.136).

## Discussion

The findings of our study denote significant differences between PPAR-alpha-deficient mice and controls in the pathology analysis of endometriotic implants. Specifically, PPAR-alpha-deficient mice seem to lack the ability to produce appropriate vascularization to sustain the same amount of endometriotic crypts in the implantation site. Concurrently, the amount of inflammation in the implantation site is significantly higher compared to control animals which may be the result of tissue necrosis due to the absence of an appropriate amount of vascularization. The amount of fibroblastic activity was significantly higher in the control group and although collagen content did not reach the level of statistical significance, one could speculate that this could be attributed to the interval that was selected between the initial auto-implantation of uterine horns and reoperation, which might be not enough to complete the process of collagen formation.

The actual impact of PPAR-alpha receptors on the process of angiogenesis has been proposed to be biphasic. Researchers support that both very high, or, in contrast, very low concentrations of PPAR-alpha may suppress tumor angiogenesis [22]. Specifically, previous studies have shown that activation of PPAR-alpha receptors with the use of agonists stimulates the angiogenic process through a gradual triggering of endothelial precursor cell activity [23]. The process seems to be mediated by inhibition of the Nod-like receptor protein 3 (NLRP3). Although to date, substantial evidence does not exist, the NLRP3 inflammasome has been indirectly related to the pathophysiology of endometriosis in several review articles [24,25].

On the other hand, inhibition of PPAR-alpha receptors also seems to reduce angiogenesis and tumorigenesis in human ovarian cancer cell lines [26]. The process seems to be exerted by a decrease in the activity of the vascular endothelial growth factor (VEGF) expression. In the experimental setting, PPAR-alpha inhibitors have been proposed as an effective treatment for peritoneal dissemination of ovarian cancer [27].

Kaipainen et al. also observed that PPAR-alpha deficiency may suppress tumor growth in an experimental murine model [28]. The process seems to be mediated by an increased inflammatory response which becomes overt in the completely deficient tissue. This inflammatory response has been investigated in the last years and researchers seem to agree that PPAR-alpha deficiency may impair normal T-cell function by inducing the expression of a high pro-inflammatory Th1 phenotype and diminishing regulatory T-cells, ultimately leading to downregulation of tumor growth [29].

In the clinical setting, resveratrol, which has been proposed to act as a potential PPAR antagonist [30], has been linked as a potential suppressor of endometriosis, an effect that is triggered through activation of the TRAIL (TNF-related apoptosis-inducing ligand) pathway [31]. The actual mode of triggering the TRAIL pathway remains unknown to date; however, researchers have previously linked the whole process with the modulatory effects of resveratrol on oxidative stress and lipid peroxidation [32].

Strengths and limitations

Our study for the first time indicates that genetically induced PPAR-alpha receptor deficiency exerts a detrimental effect on the formation and sustainment of endometriotic implants. While our findings support the findings of previous studies that indicated the potential effect of PPAR-alpha antagonists on the pathophysiology of endometriosis, they are especially important as they indicate the direct impact of PPAR-alpha deficiency (rather than downregulation) on the course of the disease. A potential limitation of our study is the relatively small number of enrolled animals, which, however, was adequate to permit the evaluation of differences that reached statistically significant levels.

## Conclusions

The findings of our study support the direct contribution of PPAR-alpha receptors in the pathophysiology of endometriosis, as the presented experimental model is free of potential medical interactions with indirect mechanisms that may link PPAR-alpha antagonists with endometriosis. To date, clinical research in this field remains extremely limited and the present experimental study may serve as a pilot for future randomized clinical trials that should assess novel pharmacological agents which may help avoid current treatment (surgical and hormonal) modalities that are associated with significant side effects.
